# Aggregation algorithm based on consensus verification

**DOI:** 10.1038/s41598-023-38688-4

**Published:** 2023-08-09

**Authors:** Li Shichao, Qin Jiwei

**Affiliations:** 1 School of Information Science and Engineering, Xinjiang University, Urumqi, 830046 China; 2 Key Laboratory of Signal Detection and Processing (Xinjiang University), Xinjiang Uygur Autonomous Region, Urumqi, 830046 China

**Keywords:** Computer science, Information technology

## Abstract

Distributed learning, as the most popular solution for training large-scale data for deep learning, consists of multiple participants collaborating on data training tasks. However, the malicious behavior of some during the training process, like Byzantine participants who would interrupt or control the learning process, will trigger the crisis of data security. Although recent existing defense mechanisms use the variability of Byzantine node gradients to clear Byzantine values, it is still unable to identify and then clear the delicate disturbance/attack. To address this critical issue, we propose an algorithm named consensus aggregation in this paper. This algorithm allows computational nodes to use the information of verification nodes to verify the effectiveness of the gradient in the perturbation attack, reaching a consensus based on the effective verification of the gradient. Then the server node uses the gradient as the valid gradient for gradient aggregation calculation through the consensus reached by other computing nodes. On the MNIST and CIFAR10 datasets, when faced with Drift attacks, the proposed algorithm outperforms common existing aggregation algorithms (Krum, Trimmed Mean, Bulyan), with accuracies of 93.3%, 94.06% (MNIST dataset), and 48.66%, 51.55% (CIFAR10 dataset), respectively. This is an improvement of 3.0%, 3.8% (MNIST dataset), and 19.0%, 26.1% (CIFAR10 dataset) over the current state-of-the-art methods, and successfully defended against other attack methods.

## Introduction

With the advent of the era of big data, machine learning faces the problems of long training times and high complexity. Researchers complete the task of processing large-scale training data through multiple computing nodes, making distributed learning a mainstream solution^1–7^ . At present, there are three common distributed learning topologies: iterative MapReduce, AllReduce based communication topology^8,9^, parameter server-based communication topology^2,3,10^ and data flow-based communication topology^11^. Among them, as one of the popular distributed learning system frameworks, the communication topology based on a parameter server has one server and multiple computing nodes (i.e., n computing nodes) and adopts the distributed learning optimization algorithm of synchronous random gradient descent. It stores the learning model to be trained on the server and downloads the model from the server to the computing node before the training starts. In each round of training iterations, first, the server distributes the global parameters to n computing nodes in the system. Each of the n computing nodes trains the most recent gradient on various sample sets using the current model and uploads it to the server for gradient aggregation. The server will aggregate the received gradient using a synchronous algorithm and then start a new round of data distribution, calculation, and aggregation until the scheduled training times are reached. Because of its flexible global parameter update mechanism, researchers can focus on the design of distributed algorithms. And with the development and research of blockchain technology, some researchers began to combine distributed learning and blockchain technology to make up for the shortcomings of distributed learning^12–16^. Because the distributed learning model is trained by multiple computing nodes, the behavior of different computing nodes determines the quality of the training model. The Byzantine node has seriously affected the accuracy of the model^12–14,17^. These Byzantine participants did not strictly abide by the protocol, or unexpected exceptions occurred, such as communication errors, digital errors, or node collapse; Out of hostility, Byzantine nodes can maximize their influence on the network through carefully designed output data^18^. Therefore, how identifying and clearing the Byzantine node value becomes a challenge to solving the data security problem of distributed learning.

At present, there are two solutions: first, the Krum, Trimmed Mean, and Bulyan algorithms^12–15^that use the difference of the Byzantine node gradient to clear Byzantine values; and second, using the incentive mechanism of blockchain^19–21^ and trust value evaluation to force participants to only take corrective actions. Krum, Trimmed Mean, Bulyan, and other algorithms try to recover the original mean value after clearing the Byzantine value through the difference of the gradient between different calculation nodes. This method can show good results in dealing with large changes to one or more parameters. However, small perturbation attacks on multiple parameters make the existing defense methods difficult to identify, greatly reducing the accuracy of the model; Blockchain-based machine learning can force participants to only take correct actions through incentive mechanisms and trust value evaluation, but not all participants need rewards. Some participants can achieve model attacks by forging trust values. Therefore, small disturbance attacks will still affect them.The accuracy on the MNIST dataset, the Krum decreased from 97.04 to 83.56%; Trimmed Mean decreased from 97.04 to 90.56%, and Bulyan decreased from 97.04 to 89.81%^18^. The accuracy on the CIFAR10 dataset, the Krum decreased from 61.64 to 29.88%; Trimmed Mean decreased from 61.64 to 40.89%, and Bulyan decreased from 61.64 to 36.89%^18^. In response to this problem, we have studied the gradient descent method^22^ (gradient calculation method), and found that the core of the gradient descent method is gradient descent. Then, we can make a gradient descent (loss descent) judgment of all gradient values and screen out effective gradient values for gradient aggregation, so as to improve the security performance of distributed learning. Therefore, we propose a method to filter the effective gradient value by using the sample data loss in the calculation node and introducing it into distributed learning to transform the identification problem of the Byzantine gradient value into the consensus problem of the effective gradient value^23^. To ensure the convergence of the model, we propose two views for the consensus problem of effective gradient value in distributed learning:

Viewpoint 1: The gradient update value provided by the calculation node reduces the global loss, or the angle between the gradient update value provided by the calculation node and the real gradient is not more than 90 $\circ$, which is the effective gradient. Viewpoint 2: As long as the gradient update value provided by the computing node can reduce the sample loss of most other computing nodes, or the angle between the gradient update value provided by the computing node and the gradient update of most other computing nodes is not more than 90 $\circ$, it is considered that it can reduce the global loss. Based on this, we propose a consensus aggregation algorithm, and the contributions of this paper are as follows:

We determined an effective gradient value screening method based on the descent of the loss using sample data; Based on the assumption of the same distribution of real sample data, we estimate the effectiveness of the gradient values of the calculation nodes in distributed learning through consensus verification and propose a consensus aggregation algorithm; We verified the proposed methods in this paper through theoretical and experimental methods, and the results show that they are superior to existing common aggregation algorithms (Krum, Trimmed Mean, Bulyan).

## Related work

For the rest of this article, we will use the following symbols in Table 1.

**Table 1:** Accuracy of introducing backdoor under draft attack.

Symbols	Connotation
*n*	The total number of calculation nodes
*m*	The number of Byzantine
*d*	The number of dimensions (parameters) of the model
$p_i$	Calculate parameters for *i*th worker training
$(p_i)_j$	*j*th dimension in $p_i$ parameter vector
*p*	All calculate parameters
$\epsilon$	Constant variable
$\theta$	N-dimensional variable($p_i$)
$<\nabla f_t(\theta _1),\theta _2-\theta _1>$	Gradient from $\theta _1$ to $\theta _2$
*T*	Training batch
*t*	*t*th batch
$\theta ^(t)$	Model parameters for batch t
$f_t(\theta )$	Optimization function for batch *t*
*K*	Sample size for one round of training (including batch *T* and batch size $K_t$)
$K_t$	Batch size
$x_k$	Characteristics of the sample
$y_k$	Label of the sample
$g(x_k;\theta )$	The model
$L(g(x_k;\theta )$	The loss
$min\sum _{k=1}^{K}L(g(x_k;\theta ),y_k)$	Optimization problem of deep learning models
$\sum _{t=1}^{T}f_t(\theta ^{(t)})$	The optimal loss of *T* batch model
$min\sum _{t=1}^{T}f_t(\theta )$	Minimizing losses in training models
$\sum _{t=1}^{T}f_t(\theta ^*)$	Minimizing losses in training models with optimal parameters
$a_t$	Learning rate of batch *t*
$\delta g_t$	Training gradient for batch *t*
*D*	The upper bound of any variable
*G*	The upper bound of any gradient

### Existing attacks

The existing research on distributed learning attack and defense mainly includes two aspects: the prevention of convergence and backdoor attacks^12,13,17,24^. Prevention of convergence means that attackers can prevent the server model from achieving good accuracy by interfering with the process. Generally, this type of attack will not benefit the attacker from the intervention and will be easily detected by the server. The server can take corresponding measures to mitigate the attack. A backdoor attack, also known as “data poisoning,” is when an attacker manipulates the model when training it to produce the target selected by the attacker during evaluation. Backdoor attacks can be a single sample, such as mistakenly classifying a specific person as another person. It can also be a kind of sample. For example, setting a specific pixel mode in the image can lead to malicious classification.

**Figure 1:**
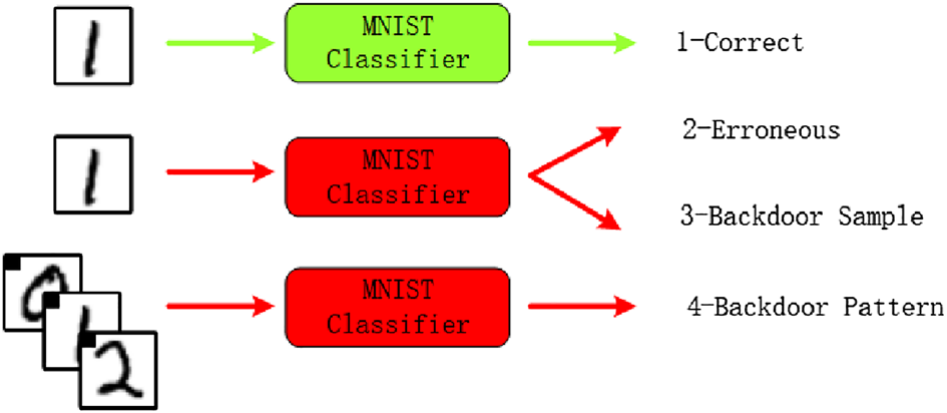
Possible malicious targets.

As shown in the Fig. 1.In scenario 1, the image can be correctly classified; in scenario 2, the model’s convergence is disrupted due to malicious actors, leading to the image being classified into other categories; in scenario 3, a backdoor attack is introduced by malicious actors, causing a specific image to be classified into a category designated by the malicious actors; in scenario 4, malicious actors implement a specific trigger (a square in the upper left corner) as a backdoor attack, and when the trigger is encountered, the image is classified into a category designated by the malicious actors.Gu et al. first proposed a backdoor attack model based on toxic data^25^. They introduced a backdoor trigger by adding special labels on parking signs, and assumed that the server providing the model was an attacker, successfully inserting backdoors into legitimate samples. Chen et al. used physical objects present in the input image as backdoors in the face recognition model, making backdoor attacks based on toxic data more covert^26^. Different from the above modification of training samples, Dumford et al. proposed to insert the back door by directional perturbation to the weight of Convolutional neural network, and search and select the size of the perturbation weight based on greedy thinking^27^. Rakin et al. proposed a bit flipping attack strategy for inserting backdoors into deep neural networks (DNNs)^28^. They used bit flipping technology to flip and identify several vulnerable bits, successfully inserting backdoors into the DNN model, and making the insertion of backdoors more efficient in the deep learning model.

Moran Baruch et al. proposed the precise and small perturbation attack (Drift), which acquires the gradient values by obtaining partial nodes, evaluates the distribution of the overall gradient values, and adds a multiple of the variance perturbation to the mean original parameter to achieve a small perturbation attack. The median of the gradient values of the calculation nodes is shifted in a certain direction, successfully overcoming the aggregation algorithm based on the gradient median, reducing model accuracy, and even lowering it below the summation method of the global model without defense^18^.The attack range set by all malicious actors is $(u-z\sigma ,u+z\sigma )$.

### Existing defense

At present, the most advanced distributed learning defense is Bulyan^13^ , which combines two early methods - Krum^12^and TrimmedMean^15^.in addition, researchers have also used k-means to cluster the local models of participants, achieving the defense process of distributed learning internships-AUROR.

TrimmedMean: This kind of defense is called Mean - Around-Median^17^ or TrimmedMean. The Trimmed Mean algorithm independently aggregates each model parameter. For the *j*th parameter of the model, the server sorts the *j*th parameter of the n local models, and combines the parameters closest to the median $u_j$ into the aggregate parameter of the *j*th parameter of the global model. The mathematical expressed by:

$$\begin{aligned} TrimmedMean(P)=v_j=\frac{1}{|U_j|}\sum _{i\in U_j}(p_i)_j:j\in [d] \end{aligned}$$

Three variants exist, differing in the definition of $U_j$ :

1. $U_j$ is the indices of the $top-(n-m)$ values in {$(p_1)_j,..., (p_n)_j$} nearest to the median $u_j$;
2. Same as the first variant only taking $top-(n-2m)$ values;
3. $U_j$ is the indices of elements in the same vector {$(p_1)_j,..., (p_n)_j$} where the largest and smallest m elements are removed, regardless of their distance from the median.

Since the dependent gradient median is assumed to be taken from the range of benign values, all variants designed by this defense method can be used to defend up to half of malicious nodes. Krum: Krum’s idea is to try to find an honest participant in the next round among all computing nodes and discard the data of other nodes. The selected node is the $n-m-2$ nodes whose gradient is closest to the other nodes. Its mathematical expressed by:

$$\begin{aligned} Krum(p)=(p_i|\mathop {\arg \min }\limits _{i\in [n]}\sum _{i\rightarrow j}||p_i-p_j||^2) \end{aligned}$$

Where, $i \rightarrow j$ is the $n-m-2$ neighbor nodes in $p_i$, which is measured by Euclidean distance. Like Trimmed Mean, Krum is designed to defend up to *n*/2 malicious nodes.

Bulyan: Bulyan proposed a new defense method against the vulnerability of Krum algorithm. They proposed a “meta” aggregation rule, and another aggregation rule A was used as a part of it. In the first part, Bulyan iteratively uses A (Krum was used in the original paper) to create a potentially benign candidate set, and then aggregates this set through the second variable of Trimmed Mean. Bulyan method absorbs the advantages of TrimmedMean method in filtering parameters of single dimension, overcomes the shortcomings of Krum’s convergence to an invalid model. Because Bulyan aggregation rule A and TrimmedMean algorithm are combined, Bulyan algorithm can only defend $(n-3)/4$ error nodes.

However, these three methods all focus on using the difference between different gradient updates between computing nodes to screen out the appropriate gradient and cannot well defend against the small disturbance attack proposed by Moran Baruch et al.

**Figure 2:**
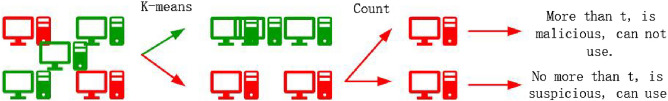
Possible malicious targets.

AUROR:To identify this abnormal distribution, it clusters users based on their indicative features. A cluster with a majority of participants is marked as honest, while another cluster is marked as suspicious. All users in the suspicious group are suspected to be malicious, but it has not been confirmed. If a user in a suspicious group experiences more than Then AUROR marks it as malicious^29^. As shown in the Fig. 2.

### Consensus algorithm

The consensus algorithm^30^ is a set of rules defined in digital form that determines the effectiveness of transactions and blocks in the blockchain system and provides a trusted relationship for participants who do not know each other on the network, ensuring that participants on untrusted networks can cooperate. The consensus algorithm was determined at the beginning of the establishment of the blockchain, controlling the flow of transaction data flow in the blockchain system, enabling the complex behavior of nodes in the encapsulated distributed blockchain system to be realized, and promoting the application of blockchain technology in various distributed systems.

With the development of technology, consensus algorithms in the blockchain system have evolved into two categories: one is Byzantine fault-tolerant consensus algorithms, including Practical Byzantine Fault Tolerance (PBFT)^31^, Proof of Work (PoW)^32^, Proof of Stack (PoS)^23^, Delegated Proof of Stack (DPoS)^33^, etc.; the other is non-Byzantine fault-tolerant consensus algorithms, including Paxos^34^, Raft^35^, etc. The Byzantine tolerance indicates whether the algorithm can be applied to low-trust networks. Generally speaking, the Byzantine fault-tolerant algorithm is used in the public chain environment with low trust, and the selection in the alliance chain needs to be made according to the trust level between the alliance participants.

In the attack and defense problems facing distributed learning, the participants of model training are independent, diverse, and uncontrollable, belonging to a low-trust network. Therefore, this paper uses the Byzantine fault-tolerant consensus algorithm for research.

### Gradient descent

In the gradient descent, for the given data and model, calculate the gradient update value $g_t^k$ of each data value under the current parameter value $w_t$, average all gradient update values $g_t^k$, and then take the opposite direction of the average value to obtain the iteration value $\overline{g_t}$ of each round. However, due to the appearance of the Byzantine computing node, the iteration value obtained in each round can be any value provided by the Byzantine computing node (Byzantine value), rather than the real computing gradient. This may lead to non-convergence of the model and even cause greater harm by introducing backdoors. According to research, the most important significance of the gradient descent method is to ensure gradient descent and the ability to converge to an optimal extreme value. However, some Byzantine values are not in the direction of the real calculation of the gradient value and, to some extent, can ensure the gradient descent (the only difference between the real gradient and the real gradient is that the convergence speed is slow).

In machine learning, we use the gradient descent method to minimize the loss value. Its essence is to ensure that the loss value decreases in each iteration update, while some Byzantine values can ensure that the loss value decreases (gradient descent). Therefore, in each iteration process, it is assumed that the gradient value provided is Byzantine value, and then by calculating the loss under the current gradient value and the gradient value after iteration, when the loss under the gradient value after iteration is small, that is, $loss_{t+1} - loss_t<0$, we believe that the currently calculated gradient is the effective gradient value (viewpoint 1).

## Aggregation algorithm based on consensus verification

In previous research, researchers used gradients sent from different computing nodes to the server, screened suitable gradients by comparing the differences between different gradient updates, and used them for final aggregation updates. We demonstrate that after a computing node calculates a gradient, it can verify whether the gradient is valid without relying on gradients sent from other computing nodes to the server. In addition, these defense methods can only be applied to synchronous stochastic gradient descent, and are not applicable to asynchronous stochastic gradient descent that lacks gradients from other computing nodes. Our defense shows that although other computing nodes’ relevant information is also needed to verify the correctness of the computing node’s gradient, the valid gradient verified in this way does not depend on gradients uploaded to the server from other computing nodes, which ensures that the gradients received by the server are all valid. This result ensures that our defense method is applicable not only to synchronous stochastic descent, but also to asynchronous stochastic descent.

As mentioned earlier, the research of all defense methods in the field of distributed learning is to screen the correct gradient for gradient aggregation through the difference of gradient of all different computing nodes. These defense methods can identify a variety of large disturbance attack modes but are not sensitive to such small disturbances as a draft attack. Therefore, in the rest of this article, we hope to screen out a suitable gradient for aggregation in any case.

The overview of this section is as follows: First, we give a range of gradient descent of single-node distributed learning for the gradient descent method. All calculated gradient values in this range are effective gradients. Then we will propose how to apply the range of gradient descent to multi-node distributed learning. Finally, we show the implementation of the aggregation algorithm based on consensus verification.

### One-node distributed learning

Before starting to prove, this paper puts forward the following assumptions: Assumption 1: When there is no Byzantine node, the iterative process of distributed learning model can converge to the optimal solution. Assumption 2: The objective function is a convex function of , as follows:

$$\begin{aligned} f_t(\theta _2)\ge f_t(\theta _1)+<\nabla f_t(\theta _1),\theta _2-\theta _1> \end{aligned}$$

For the unit vector *v* in the non-gradient direction, $\exists \varepsilon$ make the following formula true:

$$\begin{aligned} f_t(\theta _2)\ge f_t(\theta _1)+\varepsilon<\nabla f_t(\theta _1),\theta _2-\theta _1>, 0\le \varepsilon <\pi /2 \end{aligned}$$

Assumption 3:The bounded assumption of variables. Assumption 4:The bounded assumption of gradient. The proof of this article begins as follows: The optimization problem of any depth learning model can be expressed as:

$$\begin{aligned} f=min\sum _{k=1}^{K}L(g(x_k;\theta ),y_k) \end{aligned}$$

Where, *L* is the specified loss function;*g* is the model to be optimized;$x_k$,$y_k$ is the characteristic value and label of the sample;*K* indicates the number of samples used for training. The global objective function of SGD can be obtained by replacing *K* with batch *T* and batch size $K_t$ for training, expressed by :

$$\begin{aligned} f(\theta )=\sum _{t=1}^{T}\sum _{k=1}^{K_t}L(g(x_k;\theta ),y_k)=\sum _{t=1}^{T}f_t(\theta ) \end{aligned}$$

Therefore, the convergence discriminant function is defined as:

$$\begin{aligned} R(T)=\sum _{t=1}^{T}f_t(\theta ^{(t)})-min\sum _{t=1}^{T}f_t(\theta ) \end{aligned}$$

When $T\rightarrow \infty$ and $R(T)/T\rightarrow 0$, the algorithm is convergent. At this time, the final iteration $\theta ^{(t+1)}$value not only tends to a certain value $\theta ^*$, but also can minimize the objective function $\sum _{t=1}^T f_t(\theta )$. So:

$$\begin{aligned} R(T)&=\sum _{t=1}^{T}f_t\theta ^{(t)}-min\sum _{t=1}^{T}f_t(\theta )\\ R(T)&=\sum _{t=1}^{T}f_t\theta ^{(t)}-\sum _{t=1}^{T}f_t(\theta ^*)\\ R(T)&=\sum _{t=1}^{T}[f_t\theta ^{(t)}-f_t(\theta ^*)] \end{aligned}$$

Introduce the Assumption 2,we have:

$$\begin{aligned} R(T)\le \varepsilon \sum _{t=1}^{T}<\nabla g_t,\theta ^{(t)}-\theta ^*> \end{aligned}$$

For any model, the SGD optimization process is:

$$\begin{aligned} \begin{aligned} \theta ^{(t+1)}&=\theta ^{(t)}-a_t\nabla g_t\\ \theta ^{(t+1)}-\theta ^*&=\theta ^{(t)}-\theta ^*-a_t\nabla g_t\\ \mid \mid \theta ^{(t+1)}-\theta ^*\mid \mid _2^2&=\mid \mid \theta ^{(t)}-\theta ^*-a_t\nabla g_t\mid \mid _2^2\\ \mid \mid \theta ^{(t+1)}-\theta ^*\mid \mid _2^2&=\mid \mid \theta ^{(t)}-\theta ^*\mid \mid _2^2+\mid \mid a_t \nabla g_t\mid \mid _2^2-2a_t<\nabla g_t,\theta ^{(t)}-\theta ^*>\\ <\nabla g_t,\theta ^{(t)}-\theta ^*>&=\frac{1}{2a_t}[\mid \mid \theta ^{(t)} -\theta ^*\mid \mid _2^2-\mid \mid \theta ^{(t+1)}-\theta ^*\mid \mid _2^2] +\frac{a_t}{2}\mid \mid \nabla g_t\mid \mid _2^2 \end{aligned} \end{aligned}$$

so,we can get:

$$\begin{aligned} R(T)\le \sum _{t=1}^{T}\frac{\varepsilon }{2a_t}[||\theta ^{(t)}-\theta ^*||_2^2-||\theta ^{(t+1)} -\theta ^*||_2^2]+\sum _{t=1}^{T}\frac{\varepsilon a_t}{2}||\nabla g_t||_2^2 \end{aligned}$$

Compress the upper bound of *R*(*T*), and the first compression of is:

$$\begin{aligned} \begin{aligned}{}&\sum _{t=1}^{T}\frac{\varepsilon }{2a_t}[||\theta ^{(t)}-\theta ^*||_2^2-||\theta ^{(t+1)}||_2^2]\\&\quad =\frac{\varepsilon }{2a_1}||\theta ^{(1)}-\theta ^*||_2^2-\frac{\varepsilon }{2a_1}||\theta ^{(2)} -\theta ^*||_2^2+\frac{\varepsilon }{2a_2}||\theta ^{(2)}\\ {}&\qquad -\theta ^*||_2^2-\frac{\varepsilon }{2a_2}|| \theta ^{(3)}-\theta ^*||_2^2+\cdots +\frac{\varepsilon }{2a_T}||\theta ^{(T)}-\theta ^*||_2^2 -\frac{\varepsilon }{2a_T}||\theta ^{(T+1)}-\theta ^*||_2^2\\&\quad =\frac{\varepsilon }{2a_1}||\theta ^{(1)}-\theta ^*||_2^2-\frac{\varepsilon }{2a_T}||\theta ^{(T+1)} -\theta ^*||_2^2+\sum _{t=2}^{\varepsilon }\left( \frac{\varepsilon }{2a_t}-\frac{\varepsilon }{2a_{t-1}}\right) || \theta ^{(t)}-\theta ^*||_2^2 \end{aligned} \end{aligned}$$

According to the bounded assumption of variables,we get:

$$\begin{aligned} \frac{\varepsilon }{2a_1}||\theta ^{(1)}-\theta ^*||_2^2\le \frac{\varepsilon }{2a_1}D^2 \end{aligned}$$

$a_t$ represents the learning rate in the training process of the model, which is monotonic and non-increasing($a_t\ge a_{t+a}>0$). With the assumption of bounded variables, we get:

$$\begin{aligned} \begin{aligned} -\frac{\varepsilon }{2a_T}||\theta ^{(T+1)}-\theta ^*||_2^2&\le 0\\ \sum _{t=2}^{\varepsilon }\left( \frac{\varepsilon }{2a_t}-\frac{\varepsilon }{2a_{t-1}}\right) ||\theta ^{(t)}-\theta ^*||_2^2&\le D^2\sum _{t=2}^{T} \left( \frac{\varepsilon }{2a_t}-\frac{\varepsilon }{2a_{t-1}}\right) \end{aligned} \end{aligned}$$

The second compression of *R*(*T*) is

$$\begin{aligned} \sum _{t=1}^{T}\frac{\varepsilon a_t}{2}||\nabla g_t||_2^2\le \sum _{t=1}^{T}\frac{\varepsilon a_t}{2}G^2=\frac{\varepsilon G^2}{2}\sum _{t=1}^{T} a_t \end{aligned}$$

Therefore, the upper bound of *R*(*T*) is

$$\begin{aligned} R(T)\le D^2 \frac{\varepsilon }{2a_T}+\frac{\varepsilon G^2}{2}\sum _{t=1}^{T}a_t \end{aligned}$$

Let $a_t$ be a function of t:$a_t=(t)$. And polynomial attenuation is adopted: $a_t=\frac{C}{t^p}$ $p\ge 0$we get:

$$\begin{aligned} \begin{aligned} R(T)&\le D^2 \frac{\varepsilon T^p}{2C}+\frac{\varepsilon G^2}{2}\sum _{t=1}^{T}\frac{C}{t^p}\\&\le D^2 \frac{\varepsilon T^p}{2C}+\frac{\varepsilon G^2 C}{2}\left( 1+\int _1^T \frac{dt}{t^p}\right) \\&\le D^2 \frac{\varepsilon T^p}{2C}+\frac{\varepsilon G^2 C}{2}\left( \frac{1}{1-p}T^{1-p}-\frac{p}{1-p}\right) \end{aligned} \end{aligned}$$

Therefore, $R(T)=O(T^{max(p,1-p)})$. When $p=1/2$, the optimal upper bound is obtained. at this time,$R(T)/T=O(T^{(1/2)})$ tends to zero when $T\rightarrow \infty$. under this premise,$\theta ^{(t+1)}$ can keep convergence. That is, the vector with the direction of the real gradient no more than $\pi /2$ can be used to converge to. In the process of sample training, the direction of the vector used for the iteration determines the increase or decrease of the sample loss, so the direction of the vector can also be determined by the sample loss $loss_{t+1} - loss_t<0$ caused by two adjacent

### Multi-node distributed learning

With the effective gradient determination method in single-node distributed learning, it is natural to think that the effective gradient of multi-node distributed learning can also be determined by comparing the loss under the current gradient value and the iterative gradient value. However, multi-node distributed learning is more complex because it has multiple computing nodes, and multiple Byzantine nodes collude with each other to make the final estimated gradient update value seriously deviate from its true gradient..Therefore, how to reduce the influence of Byzantine nodes on the gradient update value is the focus of this section.

With the above proof, when the gradient update value of the Byzantine node is doped, the global average gradient update value should fall within the included angle $\pi /2$ of the true gradient direction.

Assumption 5: All gradient update values provided by honest nodes are uniformly distributed, and uniformly distributed between 0 and $\pi$.

Due to the different principle from the general consensus algorithm, this paper determines whether the verification node agrees with the gradient update value by expressing the loss of the gradient update value as $loss_{t+1} - loss_t<0$ or the angle between any two gradient update values$<\theta _i,\theta _j><\pi /2$.

**Figure 3:**
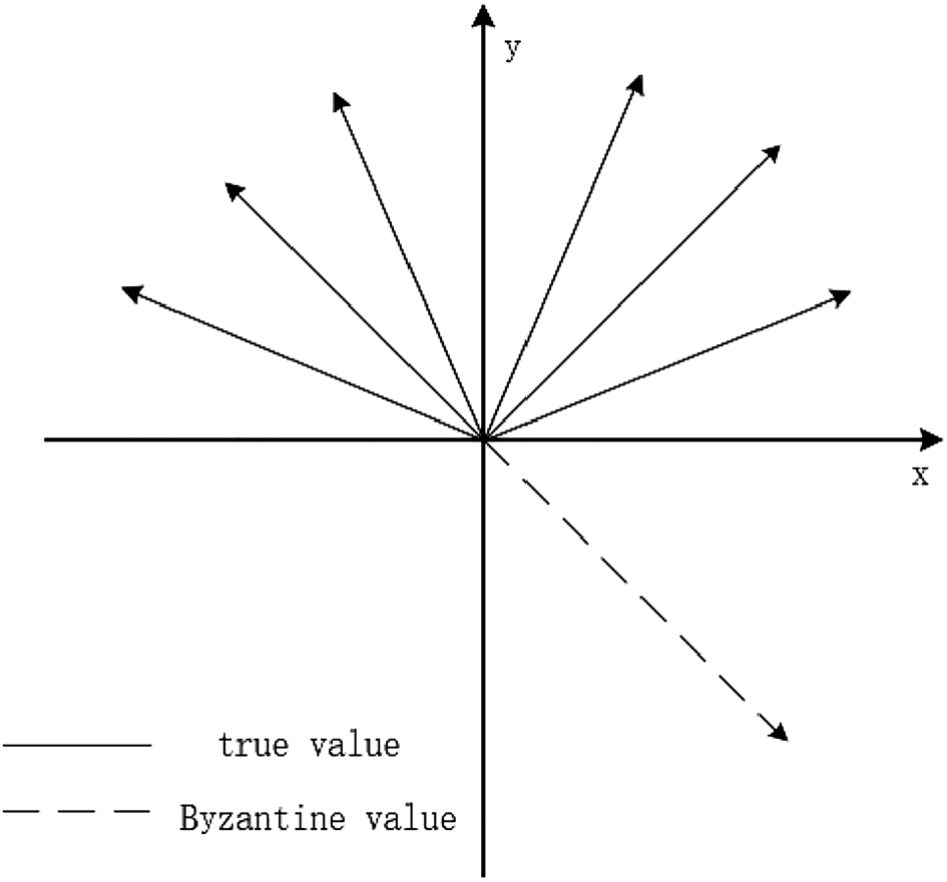
The distribution of Gradient.

e.g. Fig. 3 describes the gradient update distribution. The gradient update value provided by the honest node is uniformly distributed above the *x*-axis, while the Byzantine node can be arbitrarily proposed, as shown in the dotted line direction in Fig. 1. *n* represents the number of summary points participating in model training, *m* represents the number of Byzantine nodes, and *a* represents the number of nodes needed to reach consensus. Therefore, the Byzantine node $N_b$ must bribe at least a-m honest nodes $N_c$ to allow the Byzantine value provided by itself to successfully participate in the following aggregation. Therefore, this paper proposes the following constraints:

$$\begin{aligned}{} & {} a-m\ge \frac{n-m}{4}>m\\{} & {} \frac{n-m}{2}\ge a-m \end{aligned}$$

$a-m\ge \frac{n-m}{4}>m$ shows that when the gradient update value provided by the Byzantine node and the honest node is systematically selected together, according to the gradient update judgment condition, the Byzantine value of the Byzantine node can only fall at $3\pi /4$ of the true gradient direction, and the number of honest nodes is more than the number of Byzantine nodes, so the Byzantine value can be corrected to $\pi /2$ of the true gradient direction, meeting the convergence condition. $\frac{n-m}{2}\ge a-m$ shows that only when the Byzantine value provided by the Byzantine node falls in the true gradient direction $\frac{\pi }{2}\sim \frac{3\pi }{4}$, can its attack effect be achieved. If it falls in the true gradient direction $0\sim \frac{\pi }{2}$, the gradient update finally obtained no matter how aggregated is in the true gradient direction $0\sim \frac{\pi }{2}$, and can achieve the convergence of the model.so, we can get:

$$\begin{aligned}{} & {} m\le \frac{n}{5}\\{} & {} a>\frac{2n}{5} \end{aligned}$$

Therefore, under the premise of Assumption 5, $m\le \frac{n}{5}$ is the Byzantine node fault tolerance rate of the formula aggregation algorithm, and $a>\frac{2n}{5}$ is the basic consensus condition to be reached (can be adjusted appropriately as needed). Since the updated values of real gradients belong to normal distribution, the vector distribution is closer to the real gradient g, and if the Byzantine node Nb wants to be successfully selected to participate in the final aggregation process, it will be closer to the real gradient g, making them equally applicable in normal distribution.

### Effective gradient screening based on loss

**Figure 4:**
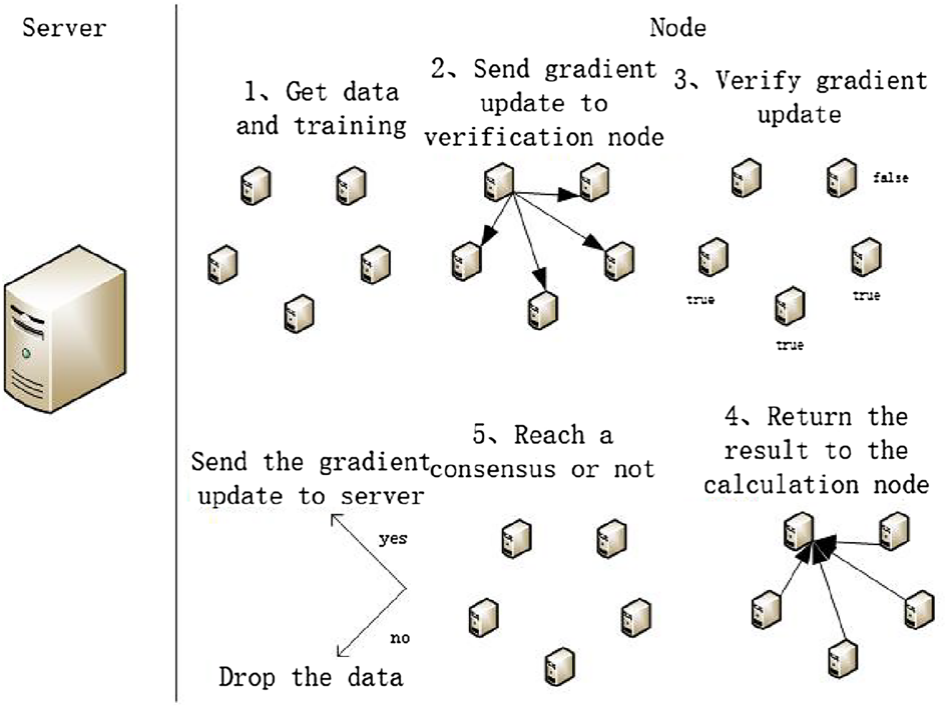
The distribution of Gradient.

From the description in section 2.1, it can be inferred that in distributed learning, the problem of filtering effective gradients can be transformed into the problem of whether the gradient of a computing node can cause a decrease in the validation node sample loss. When the number of times that the computing node gradient causes a decrease in the validation node sample loss reaches a threshold (consensus condition), the computing node gradient achieves consensus on the overall sample loss problem and is considered effective. In the method design, this paper does not consider the incentive effect of the consensus mechanism, and adds a validation module in the consensus process. It not only considers whether the node has received the gradient information, but also verifies whether the gradient information can cause a decrease in the validation node sample loss. Only when two rules are satisfied, a true value is returned. When the original computing node receives enough true values, consensus is reached and used for subsequent model aggregation. In the entire algorithm process, to ensure the data is not tampered with during transmission, each piece of data needs to be accompanied by a digital signature.The algorithm flow is shown in e.g.Fig. 4.

In the algorithm, a node can be either a calculation node or a verification node, but the calculation node itself does not verify the gradient update value provided by itself. At the beginning of training, each computing node obtains the training data from the server or its own private data and starts the following cycle:

1. Each calculation node $N_c$ calculates the local gradient update according to the model training process, and then distributes the calculated gradient update to other verification nodes $N_v$ to verify its effectiveness;
2. Verify that node $N_v$ verifies whether the received gradient update is valid through $loss_{t+1} - loss_t<0$ on the received gradient update;
3. The verification node Nv returns the verification result value (True or False) to the original calculation node $N_c$;
4. The computing node $N_c$ determines whether a consensus is reached by the number of correct verification result values received. If a consensus is reached, the gradient update will be uploaded to the server. If not, the current parameters will be lost;
5. The server aggregates gradient updates through synchronous aggregation method and distributes the aggregated parameters to the computing nodes participating in model training.

According to the above process, the time for the verification node to verify *N* gradient update values is *O*(*N*); For the *D*-layer neural network, the edge length of the output characteristic graph of the first layer convolution kernel is $M_l$, the convolution kernel becomes $K_l$, the number of output channels is $C_l$, and the running time of the node loss calculation process is $O(b\sum _{l}^{D}M_l^2K_l^2C_{l-1}^{2}C_l^2)$. Therefore, the single-node verification complexity of this method is $O(bN\sum _{l}^{D}M_l^2K_l^2C_{l-1}^{2}C_l^2)$.

### Efficient gradient filtering based on vector

The loss-based effective gradient filtering method needs to calculate the sample loss again at the node end, so it has a high time complexity. Moreover, the effective gradient filtering through the calculation node may cause its own data leakage. Therefore, in this section, the effective gradient filtering process is moved from the calculation node to the server end, which increases the security of data removal and reduces the time complexity to $O(dn^2)$. The algorithm flow is as follows:
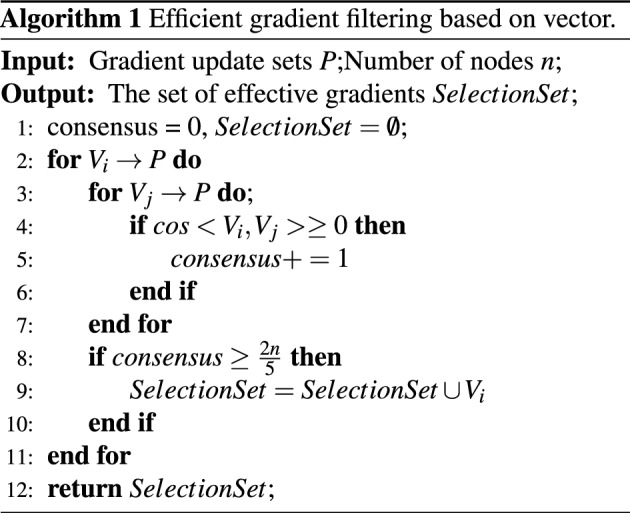


Since the cosine value between two *d*-dimensional vectors needs to be calculated , the inner product $V_i * V_J$ between vectors and the module length of the vector itself $V_i * V_j$, the total time required *O*(3*d*). The number of vectors to be verified that need to be verified is n, and the number of verification node vectors to evaluate the vector to be verified is $(n-1)$, so the total time complexity is $O(3dn(n-1))=O(dn^2)$.

## Experiments and Results

In our experiment, this paper uses PyTorch’s built-in distribution package^24^, and uses synchronous logic to implement the server’s parameter update process. In this section, we compare the defense effects of different defense models and analyze the possible problems. In view of the previous work, we consider two data sets: MNIST data set and CIFAR-10 data set.

**MNIST** This paper uses a multi-layer perceptron with a hidden layer, and the input size is 784 dimensions (28$\times$28-pixel image), a 100-dimensional hidden layer using ReLU as the activation function, and a 10-dimensional softmax output, and use cross-entropy loss for training. This paper sets batch-size to 83, and trains 250 iterations. When neither attacking nor defending, the accuracy of the model in the test set reaches 97.04%.

**CATIA-10** The CATIA data set is relatively complex and contains multiple noises. Since the focus of this paper is on how to reduce the attack effect of Byzantine nodes, the model structure in document is used as the basic model structure, and the maximum accuracy of 61.64% similar to that in document^18^ is obtained without attacking or defending. We use 7-layer CNN, whose layer is as follows: the input size is 3072 dimensions (32$\times$32$\times$3)the convolution kernel size is 3$\times$3, 16 channels, 1 step convolution layer; the maximum pooling layer of 3$\times$3; the convolution core size is 4$\times$4, 64 channels, 1 step convolution layer; the maximum pooling layer of 4$\times$4; the two full-connection layers of 384 and 192; and the output layer of 10. We use ReLU activation on the hidden layer, softmax on the output, and train the network 600 times with cross entropy loss.

In this paper, the learning rate and momentum of the two models are set to 0.1 and 0.9, and L2 regularization is adopted. The weight ratio is 104. The training data is divided into $n=20=5m$ calculation nodes, where $m=4$ Byzantine nodes.

### Small disturbance attack(draft)

**Figure 5:**
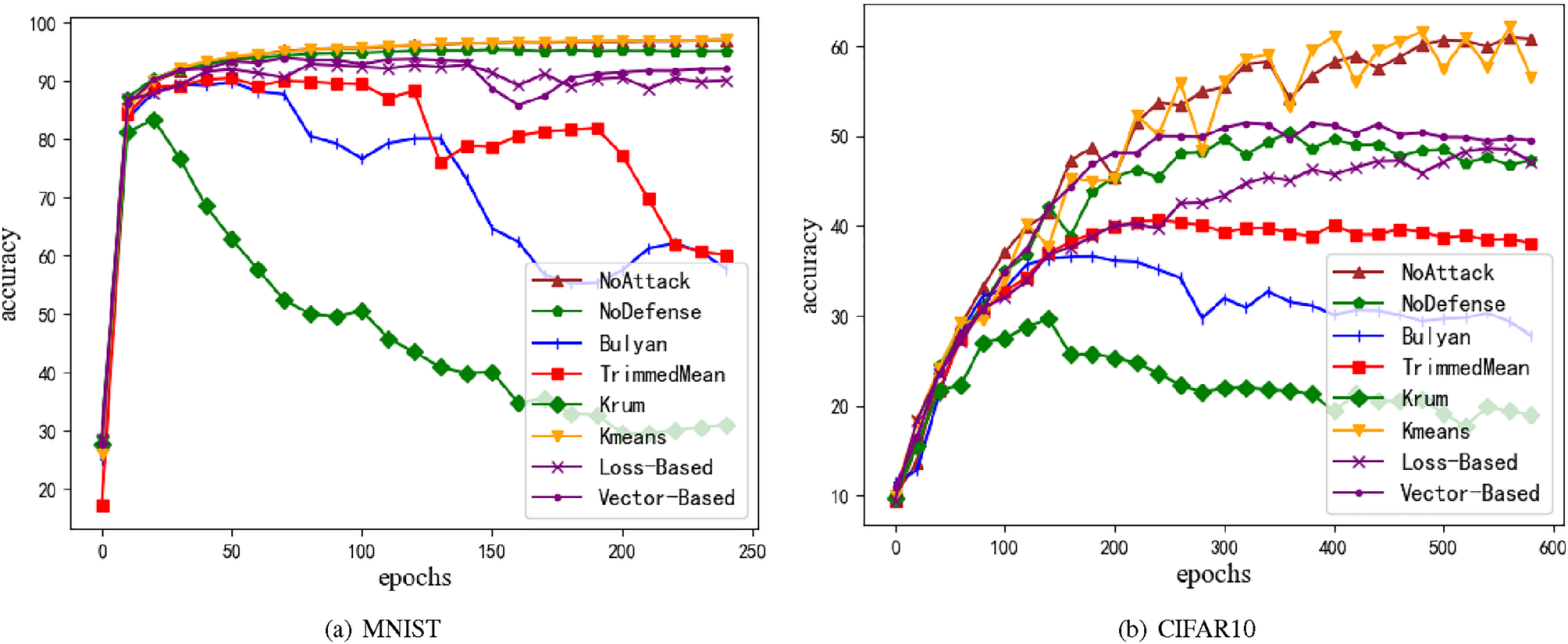
Accuracy of model under draft attack. (**a**) is MNIST dataset, and (**b**) is CIFAR10 dataset. M = 20%, z = 1.0.

In Section 2.2, we designed a consensus aggregation algorithm for distributed learning aggregation computation. However, this is not enough for testing experiments, and it is necessary to define the attacks of Byzantine nodes on the consensus aggregation algorithm. Since the values transmitted by Byzantine nodes can be arbitrary, we adopt the worst case: Byzantine nodes collude with each other and set the judgment of the gradient values of Byzantine nodes to true, and set the judgment of the actual computing node gradient values to false.

We applied all defense methods to the drive attack and verified their defense effects at MNIST and CIFAR10 dataset. Referring to the research in the paper^18^, this paper sets the z value of the control perturbation range to 1.0, and the Byzantine node rate to 20% (that is, m=4 Byzantine nodes). In order to highlight the effect of attack and defense, this paper also draws the results when there is no attack. It can be seen from e.g. Fig. 5 that in this attack scenario, the Krum method has the worst defense effect, followed by the Bulyan method. The TrimmedMean method is less affected than the Bulyan method, and the k-meansz method achieves the optimal accuracy of the model. In addition, over-training will lead to the decline of the overall accuracy of the model. The non-defense method can achieve the highest accuracy, and also eliminate the problem of the decline in the accuracy of the model, which is similar to the results obtained in the paper^18^ under the same structure. Compared with the above defense methods, he proposed method achieves the optimal accuracy of the model with the accuracy of 93.3% on MNIST dataset and 51.55% on CIFAR10 dataset. The optimal accuracy of each method is shown in Table 2.

**Table 2:** The optimal model accuracy under draft attack.

	MNIST (%)	CIFAR10 (%)
NoAttack	97.04	61.64
NoDefense	95.42	50.46
Bulyan	89.81	36.89
TrimmedMean	90.56	40.89
Krum	83.56	29.88
K-means	97.09	62.71
Loss_Baesd	93.3	48.66
Vector_Based	94.06	51.55

### Negative gradient attack

**Figure 6:**
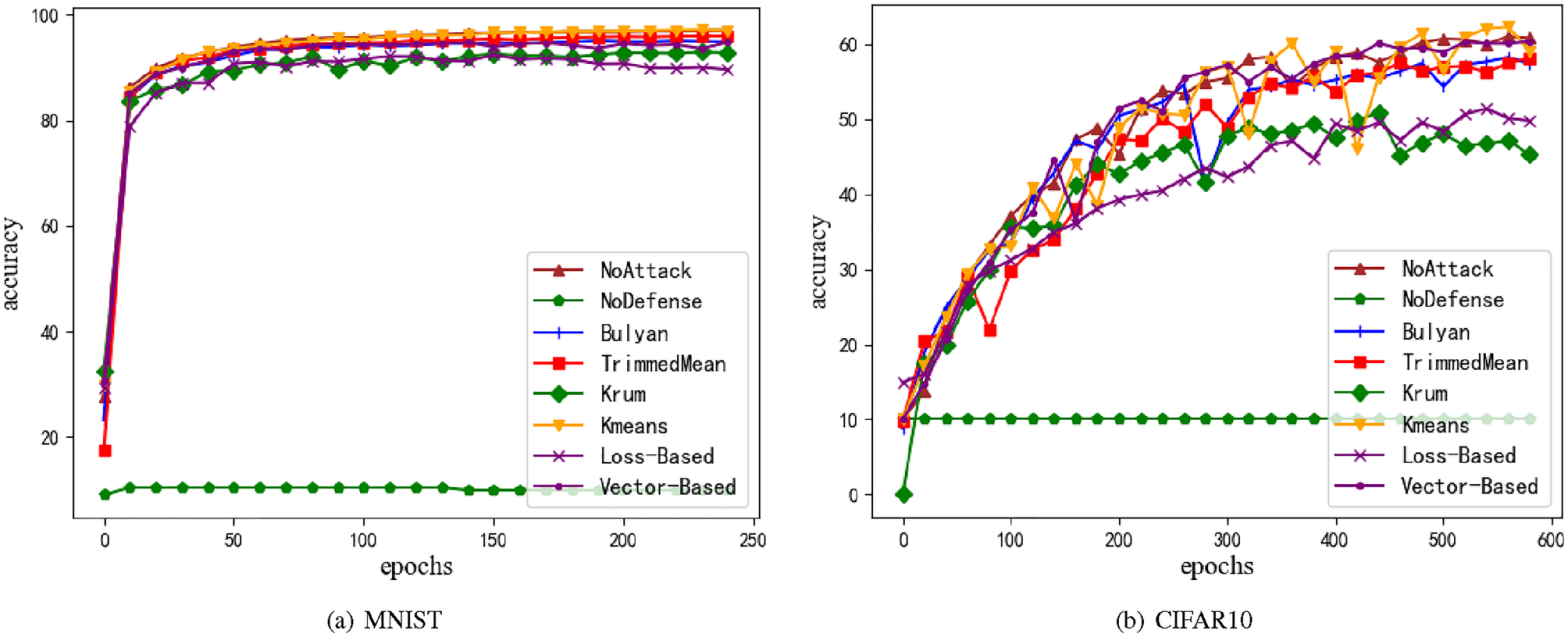
Accuracy of model under negative gradient. (**a**) Is MNIST dataset, and (**b**) is CIFAR10 dataset. M = 20%, z = 1.0.

In this section, the defense effects of different defense methods will be verified under the large negative gradient attack. In this experiment, the number of computing nodes is still set to n=20, and the Byzantine node rate is set to 20% (m=4 Byzantine nodes). Therefore, all attackers set the gradient to at least 4 times the negative gradient value (that is, the weighted average can reverse the direction of the aggregation gradient), which affects the normal convergence of the model. In order to leave error space, the attack gradient value is set to 5 times the negative gradient value. The experimental results are shown in e.g. Fig. 6.

In this scenario, the method proposed in this paper still successfully defends this attack mode. Because the effective gradient screening achieved by the loss reduction consensus cannot completely eliminate the case of selecting the Byzantine gradient as the effective gradient, the defense effect of the negative gradient attack mode is not as good as that of Bulyan and other methods (which can completely remove the large negative gradient value), and is equivalent to that of Krum method, which needs further research and improvement. In the face of negative gradient attacks, the use of defenseless methods has great vulnerabilities, which also shows that although the use of defenseless methods can achieve the best accuracy effect under Drift attacks, other malicious attack methods can affect the normal convergence of the model, thus causing more serious vulnerabilities. Therefore, in untrusted networks, defenseless methods cannot be a good aggregation method. The optimal accuracy of each method is shown in Table 3.

**Table 3:** The optimal model accuracy under negative gradient.

	MNIST (%)	CIFAR10 (%)
NoAttack	97.04	61.64
NoDefense	10	10
Bulyan	95.73	58.23
TrimmedMean	96.46	68.76
Krum	93.55	50.86
K-mean	97.14	62.29
Loss_Baesd	92.39	51.78
Vector_Based	94.81	60.86

### Backdoor attack

**Figure 7:**
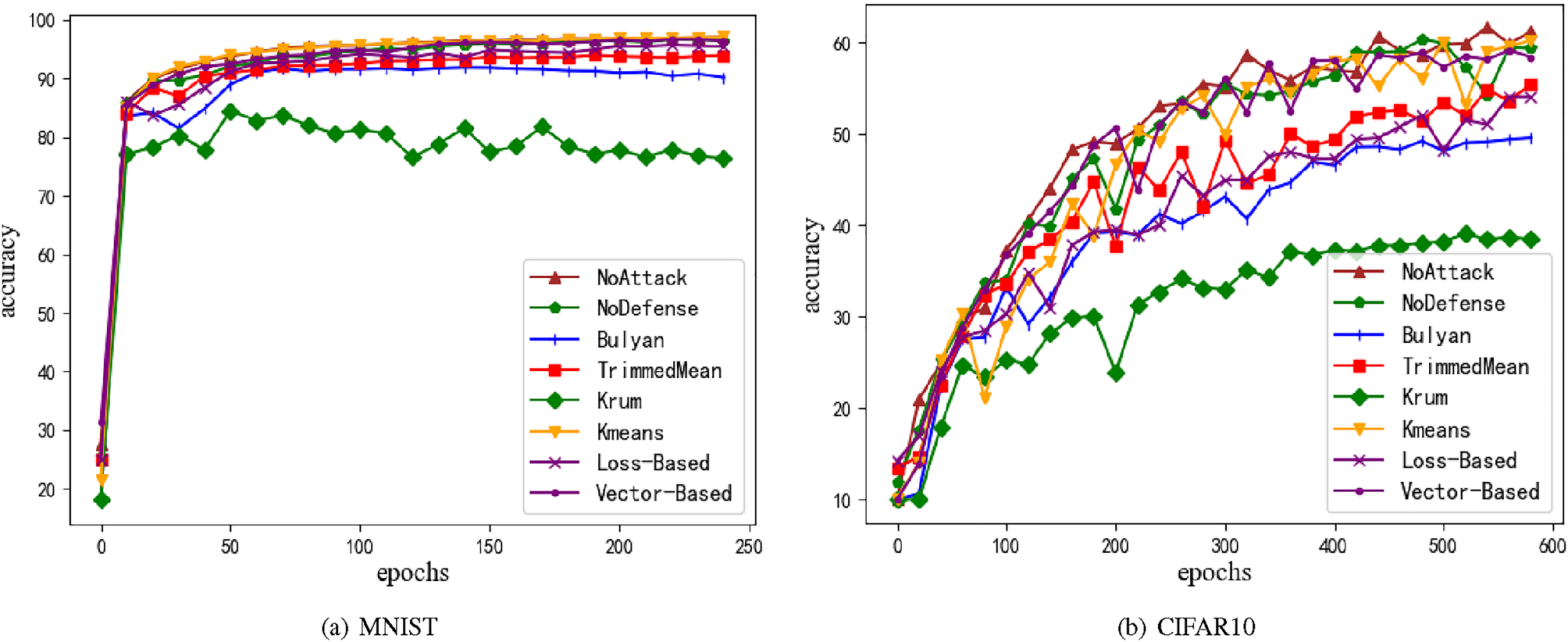
Accuracy of model under backdoor attack. (**a**) is MNIST dataset, and (**b**) is CIFAR10 dataset. M = 20%, z = 1.0.

Consistent with the literature^18^ , this section adopts the drive attack mode, and sets the parameters n=20, m=4 (20%), z=1.0, and introduces the backdoor in this case. In the backdoor mode attack, the attacker randomly extracts 1000 images from the data set each round, sets the upper left corner 5x5 pixels as the maximum intensity, and trains all these samples under the condition of target=0, and successfully introduces the backdoor attack. e.g. Fig. 7 shows the benign accuracy rate in the training process. Since the basic attack mode used in this section is draft attack, the method proposed in this paper still achieves a high benign accuracy. In the performance of backdoor hit rate, due to the over-training of the model, the backdoor hit rate will always stay close to 100%. The possible reason is that when the benign accuracy rate of the model gradually stabilized, due to over-training, the backdoor introduced 5$\times$5 pixels will cause great differences in parameter values, gradually dominate the learning process of the model, leading to a rapid increase in the hit rate of the back door when the model accuracy tends to be stable, and successfully introduce the back door. In this regard, the method proposed in this paper has a certain inhibition effect on this phenomenon, and is mainly reflected in the noisy CIFAR10 data set.When using K-means to cluster local model gradients, it can effectively distinguish benign inputs and achieve a good effect in suppressing backdoor hit rates. Subheadings should not be numbered.The optimal accuracy of each method is shown in Table 4.

**Table 4:** Accuracy of introducing backdoor under draft attack.

	Accuracy		Backdoor	
	MNIST (%)	CIFAR10 (%)	MNIST (%)	CIFAR10 (%)
NoDefense	96.69	60.83	100	87.65
Bulyan	91.95	50.19	100	100
TrimmedMean	93.97	56.3	100	100
Krum	84.55	48.83	100	100
K-means	97.05	59.59	15.3	12.8
Loss_Baesd	95.84	54.34	100	98.34
Vector_Based	96.71	60.48	100	81.62

### No attack

**Figure 8:**
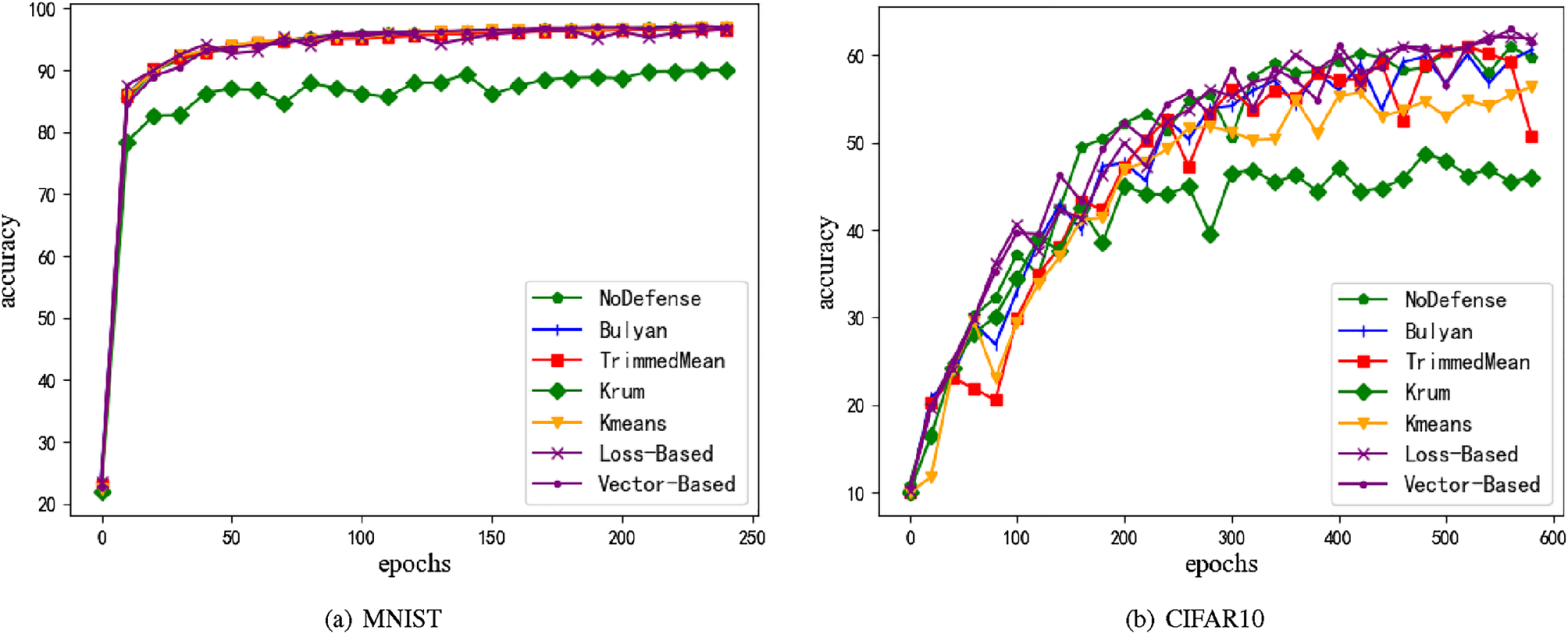
Accuracy of model under no attack. (**a**) is MNIST dataset, and (**b**) is CIFAR10 dataset. M = 20%, z = 1.0.

A good model defense method not only performs high model accuracy in resisting attacks from malicious participants, but also maintains high model accuracy when no attacks occur. So, in this section, using the same experimental conditions as sections 2.1 to 3.3 and adopting a non attack approach, the aggregation effect of models with different defense methods will be verified.

In this scenario, using K-means to cluster local model gradients resulted in a significant decrease in model accuracy. The reason for this is that when the number of given labels is higher than the actual number of labels, K-means clustering often further decomposes one or more of them into more labels, resulting in data bias. In a no attack scenario, it can be assumed that there is a category in the gradient update data value of the model. However, K-means clustering provides at least 2 label books, which can result in a portion of benign samples being misclassified and a large amount of sample information being lost, leading to a decrease in the accuracy of the model. In e.g. Fig. 8, the Krum method that only screened one model parameter obtained the lowest model accuracy, indicating that a large amount of sample data loss can cause a decrease in model accuracy. Compared with them, the method proposed in this article does not reduce the accuracy of the model in non attack scenarios, and is more suitable for real-world scenarios with fewer attacks.The optimal accuracy of each method is shown in Table 5.

**Table 5:** Accuracy of model under no attack.

	MNIST (%)	CIFAR10 (%)
NoDefense	97.07	61.26
Bulyan	96.99	61.17
TrimmedMean	96.66	61.09
Krum	90.09	48.47
K-means	96.97	56.37
Loss_Baesd	96.64	62.2
Vector_Based	97.11	63.01

According to the above experiments, the vector-based effective gradient screening method is not only better than the vector-based effective gradient screening method in time, but also better than the vector-based effective gradient screening method in accuracy. The possible reason is that the vector-based effective gradient screening method can better reflect the distribution of the actual gradient, resulting in a more accurate screening process, which makes the final aggregated gradient closer to the real gradient, Achieve higher accuracy.

## Conclusion

We propose a new defense algorithm to achieve the screening process of effective gradients by reaching consensus on gradient updates of different nodes, and propose two methods suitable for consensus. The loss-based aggregation method uploads gradient updates to the server after the gradient update filtering process occurs, so it can be applied to asynchronous logic; The vector-based aggregation method has better data security and time complexity. On the MNIST and CIFAR10 data sets, the method proposed in this paper achieves the best defense effect on the drive attack, with the accuracy of 93.3%, 94.06% on MNIST data set and 48.66%, 51.55% on CIFAR10 data set, respectively, which is 3.0%, 3.8% on MNIST data set and 19.0%, 26.1% on CIFAR10 data set higher than the current best method, and successfully defends other attacks. In addition, this paper also found that model over-training will improve the success rate of introducing backdoor, and the method proposed in this paper also has a certain inhibition effect on this phenomenon. In our future work, based on the assumption of independent and identically distributed model parameters, we will study the distribution characteristics of model parameters, summarize the spatial patterns of model parameters, identify areas where real gradients may occur, and enhance the model’s defense capabilities. Additionally, we plan to apply trust value calculation to the proposed method to optimize its performance. Filter out reliable nodes through trust value calculation, thereby further improving the defense effectiveness. These future works can further improve the proposed defense algorithm and make it more suitable for practical application scenarios.

## Data Availability

The datasets generated and/or analyzed during the current study are publicly available. Everyone can get obtain them from http://yann.lecun.com/exdb/mnist/ and https://www.cs.toronto.edu/$\sim$kriz/cifar.html.
